# The First Dorsal Metacarpal Artery Flap Family: A Review

**DOI:** 10.1055/s-0038-1675369

**Published:** 2018-10-29

**Authors:** Jose Couceiro, Marta de Prado, Guillermo Menendez, Zaira Manteiga

**Affiliations:** 1 Hand Surgery Unit, Orthopedics Department, Hospital Marques de Valdecilla, Santander, Ca, Spain; 2 Orthopedics department, Hospital Lucus Agusti, Lu, Spain

**Keywords:** first dorsal metacarpal artery, FDMA flap, thumb trauma, thumb recosntruction

## Abstract

Deep soft tissue injuries around the thumb can severely hinder hand function if not treated correctly. Many different surgical options have been described for the treatment of these serious lesions, including microsurgical flaps, such as toe to hand flaps, local flaps, and distant pedicled flaps. The first dorsal metacarpal artery family of flaps belongs to this last category. These flaps can be performed in different ways, as a racquet, as an island flap, and as a bilobed flap including the second metacarpal artery, among others.

The aim of the following article is to review the basic concepts involved in the use of these flaps in reconstructive hand surgery.

The first dorsal metacarpal artery (FDMA) family of flaps consists of several different pedicled flaps which rely on the use of the FDMA, alone or in combination with the second dorsal metacarpal artery. 1 2 3 4 5 6 7 8 These flaps include the island flaps, racquet flaps, and bilobed flaps among others. The use of the aforementioned flaps has been extensively reported in the indexed literature, mainly for the repair of deep soft tissue defects of the thumb.

The current article reviews the surgical anatomy, techniques, tips, tricks, and expected results when using these complex yet versatile flaps.

## Surgical Anatomy

**Fig. 1:**
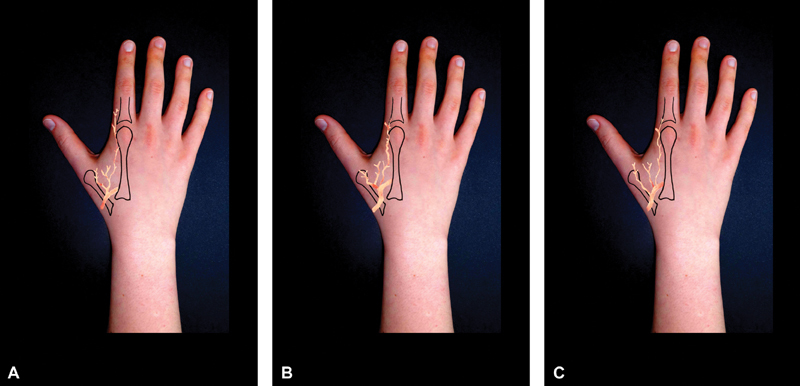
The three different patterns of the first dorsal metacarpal artery. Type I, **(A)** appears to be the most common one, with three vessels arising from a common trunk. Types II **(B)** and III **(C)** are far less common.

The first dorsal metacarpal artery has been found to be quite constant. Sherif 1 differentiated three different vascular patterns for the terminal fascial branches of the FDMA ( Fig. 1 ). Type I was the most frequent (11 out of 18 specimens), with three vessels, ulnar, intermediate, and radial originating from a common trunk that arose from the radial artery. Type II was far less frequent (3 out of 18 specimens) it involved two vessels originating from a common trunk, and one separate vessel issuing from the radial artery. On type III (4 out of 18 specimens), three arteries originated directly from the radial artery.

Murakami et al 2 reported the presence of these three arteries as well in all of their cadaveric dissections.

Most of the flaps on the FDMA family are based on the ulnar branch (FDMAu). This ulnar branch ends as a vascular plexus over the dorsal fascia of the index finger. It is important to note that a distal perforator is frequently present at the radial side of the index finger at the height of the metacarpophalangeal joint, this distal perforator connects the FDMAu to the second palmar metacarpal artery and must be generally either ligated or coagulated to perform the flap.

The FDMAu is typically accompanied by one or two veins, 1 these provide the venous outflow which is extremely important for the procedure.

**Fig. 2:**
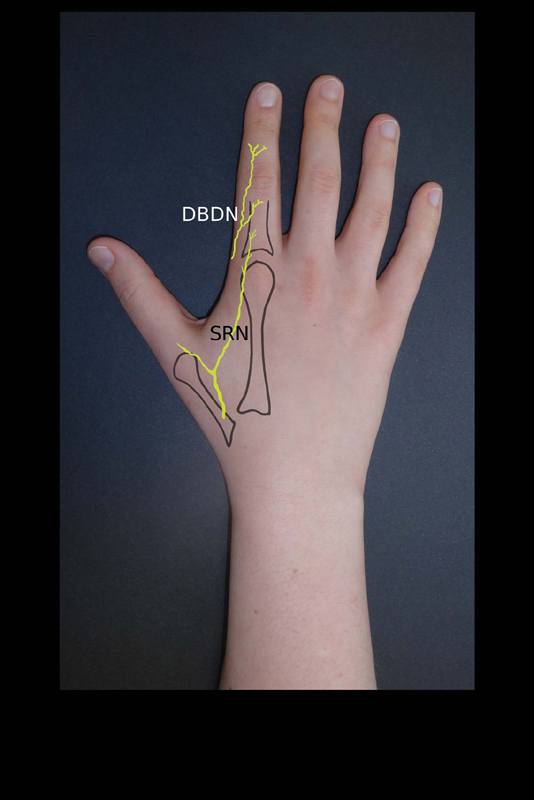
The flap is innervated by sensory radial nerve branches (SRN), the dorsal branch of the digital nerve (DBDN) can be harvested with the flap if additional innervation is desired.

According to Tellioglu and Sensöz, 3 the terminal sensitive branches of the radial nerve are present at the dorsum of the index finger, at the area of the proximal phalanx, lying just beneath the skin. In their dissections they always found a dorsal branch of the digital nerve ( Fig. 2 ) which was deeper than the radial nerve branches, lying just over the extensor apparatus, and ending as three terminal branches on the area of the middle phalanx. This dorsal nerve branch can be sacrificed to add innervation to the flap if necessary. 4 5

## Indications and Contraindications

The major indication for the use of the FDMA flaps is the presence of deep soft tissue injuries around the thumb. 6 The FDMA flaps have also been used to restore thumb sensibility 7 and to treat cold intolerance. 6 8 Foucher and Braun mention the use of this flap to treat venous congestion in replanted thumbs as well. 8

Active thumb infections, trauma to the index finger affecting the traject of the artery, and an absence of a preoperative Doppler's signal are all contraindications for the use of these flaps.

## Surgical Technique

**Fig. 3:**
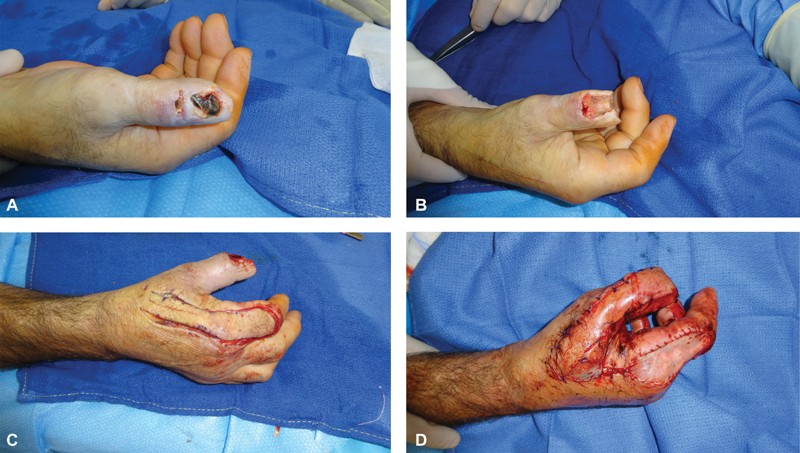
Intraoperative pictures, on this case the patient had sustained a crush injury to his left thumb **(A)** , after the resection of the necrotic areas **(B)** , an FDMA racquet flap is raised **(C)** and used to cover the defect. FDMA, first dorsal metacarpal artery.

The surgical technique for all of these flaps is commonly performed with magnification loupes and under ischemia provided by a pneumatic tourniquet ( Fig. 3 ). A video of the dissection of the flap by the senior author can be found at the *vumedi* web ( *https://www.vumedi.com/video/how-to-perform-a-holevich-flap/* ).

### Island Flap

To perform the FDMA flap as an island, 8 the first step is to identify the artery with a Doppler's probe, the path is then marked with a needle. The flap is outlined on the dorsum of the index finger, the width and length of the flap must match the thumb defect. The dissection is started ulnarly and distally. Great care is taken not to injure the peritenon when raising the distal part of the flap. A lazy **S** incision is performed parallel to the radial border of the second metacarpal, a fascial strip containing the FDMAu and two veins are harvested with the flap. The dissection stops proximal to the radial artery which is the pivot point.

On the distal radial side of the index finger, it is advisable to include a small part of the extensor hood with the flap, this is meant to prevent injuries to the vascular connection between the skin island and the vascular pedicle. The distal perforator must be either ligated or coagulated away from the flap at this level. When performing the radial dissection, it is possible to incorporate the dorsal nerve branch from the collateral nerve to the flap, creating an innervated FDMA flap. This nerve branch is typically sutured to another branch on the operative field in an end-to-end fashion.

Once the dissection is completed the flap is transposed to the thumb, crossing under a skin bridge, it is recommendable to keep this tunnel under the skin loose, so as to lessen any compressive effects on the vascular pedicle.

### Racquet Flap

The racquet flap technique is very similar to that of the island flap. 6 7 The path of the artery is marked with the Doppler's probe and the dissection is identical to that of the island flap; except for the area of the vascular pedicle. When performing the racquet flap, not only the fascia but also a skin paddle is included, this paddle is around one centimeter wide and it is centered longitudinally on the FDMAu. When transposing the racquet flap, an incision is made along the desired path on the thumb, there is no need for a skin bridge or tunnel in this technique.

The racquet flap is technically less demanding than island flap, it obviates compressive areas and the venous outflow is probably better. 6 The island flap may provide a slightly better aesthetic outcome.

### Bilobed Flap

**Fig. 4:**
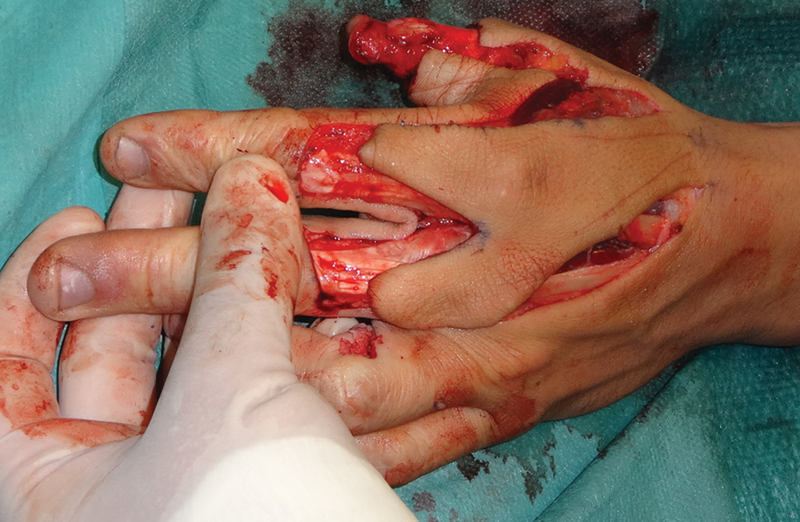
A bilobed racquet flap, in this case, an extended seagull flap as described by Couceiro et al. 10

The bilobed flap is a more challenging flap, it is meant for the coverage of very large thumb defects, or as a salvage procedure following the unsuccessful replantation of a degloved thumb. It can be executed either as a racquet or as an island flap. 9 10 The paths of the first and second metacarpal artery are identified with a Doppler's probe and marked with a needle. The dissection is once again started ulnarly, the area on the dorsum of the middle finger and a small section of skin connecting the index and middle finger are included as well. Great care is taken not to injure the peritenon of both digits when raising the flap. Proximally, one can proceed two ways, one with a lazy **S** incision, raising a fascial strip containing both neurovascular bundles with the accompanying veins and branches of the radial nerve or raising a skin paddle incorporating all of the aforementioned structures. If the fascial strip is chosen care must be taken to have a tunnel of sufficient size to accommodate both pedicles; if the flap is executed as a racquet a skin incision must be performed on the thumb to accommodate the skin. ( Fig. 4 )

There are some FDMA flap variations that may be used for special applications. El-Khatib 11 described an extended flap that included not only the skin on the dorsum of the proximal phalanx of the index finger but also the one overlying the proximal interphalangeal joint and middle phalanx.

Couceiro et al 10 described the use of an extended seagull flap for the coverage of a thumb degloving injury, they included a very big skin paddle and the skin on the dorsum of the proximal phalanxes of the index and middle finger.

Chen et al 5 reported the use of an innervated FDMA flap which included both, the ulnar and radial dorsal branches of the proper digital nerves, they concluded that this modification was useful for the restoration of sensation on the thumb tip.

## Tips and Tricks

Racquet flaps are usually considered less technically demanding, the venous outflow is assured by the proximal skin area.

Island flaps involve a slightly more complex procedure, care must be taken when preparing the tunnel under the skin as it must accommodate the pedicle with minimal or no compression.

A key region in the dissection is the radial border of the second digit, the perforator at this area is quite constant and must be coagulated or ligated away from the flap. It is important to resect a piece of the extensor hood with the flap.

When executing a racquet flap it is important not to dissect the skin on the paddle from the underlying fascia. Unless an innervated flap is planned do not resect or injure the dorsal branch of the radial collateral nerve. Marking the path with a needle is one of the authors personal preferences, skin markers may be erased when prepping the patient for surgery.

## Outcomes

### Sensibility

Cortical reorientation has been reported to be absent in up to 52% 4 of the cases, this does not frequently affect the patient's activities of daily living.

Two tip discrimination for this flap is commonly around 9 to 10 mm. 4 6

### Index Finger and Thumb Range of Motion

Zhang et al 13 Report a similar range of motion for the thumb and index metacarpophalangeal joints, for the proximal interphalangeal joint of the index, and the interphalangeal joint of the thumb when compared with the contralateral side.

Couceiro and Sanmartín 6 publish similar findings, five of their patients had a 15-degree flexion deficit at the interphalangeal joint of the thumb.

Muyldermans and Hierner 12 had a mean 7.41 Kapandji's score for the thumbs in their case series.

### Patient Satisfaction

Satisfaction in the absence of complications appears to be quite high, 6 care must be taken with aesthetics in female patients and patients with special concerns.

## Pitfalls and Complications

### Necrosis and Venous Congestion

The expected percentage of flap necrosis is low for FDMA flaps, Zhang et al 13 reports a partial necrosis of 2 of their 42 flaps in their case series, Couceiro and Sanmartín 6 report a partial necrosis of 2 of their flaps in a 10 flaps case series, 5 of the island flaps and 5 of them racquet flaps.

El-Khatib 11 reports experiencing venous congestion in all of the five flaps in his case series, Zhang et al 13 refer experiencing some degree of venous congestion on their case series, two of the island flaps on Couceiro and Sanmartín case series 6 experienced venous congestion, the authors report finding a higher degree of venous congestion and flap necrosis on the island flap group, their findings were not completely conclusive owing to the limited size of their study.

If venous congestion is present after performing an island flap this may be salvageable revising and widening or opening the tunnel to the thumb.

### Cold Intolerance

twenty percent of patients experience cold intolerance on their thumbs. 4 It has been discussed that this may be slightly lower for racquet flaps. 6

### Donor Site Morbidity

Patients appear to have few problems with the donor site; however, Trankle et al 4 on their series of innervated FDMA flaps referred an 8% (two patients) of cold intolerance at the donor finger, a 16% (four patients) diminished protective sensibility at the donor area, and a diminished sensibility at the radial side of the index finger in 4% (one patient), the total range of motion of the index finger was diminished by 4.4%.
